# Must We Use ASI in the Nordic Region?

**DOI:** 10.1177/14550725261502835

**Published:** 2026-10-30

**Authors:** Jørgen G. Bramness

**Affiliations:** 1 Department of Clinical Addiction Research, 155272 Oslo University Hospital, Oso, Norway; 2 UiT The Arctic University of Norway, Tromsø, Norway; 3 Research Center for Substance Use Disorders and Mental Illness, Innlandet Hospital Trust, Hamar, Norway

The Addiction Severity Index (ASI) and its European adaptations are structured multidimensional assessment instruments designed to evaluate the severity of substance use disorders (SUDs) and associated difficulties in major life domains, thereby supporting treatment planning, outcome evaluation, and research (European Union Drugs Agency, 1980).

The opinion piece by Alexander Björk questions the technical quality and most of all usability of the ASI interview, based on the Swedish authority's recommendation that the ASI interview *should* be used in the country's clinical addiction services (Björk, 2026). This contrasts with the recommendations in Norway and Finland where the recommendation is *may* be used and Denmark, where no recommendation is fronted. The Swedish version of ASI is adapted from the original version of ASI (McLellan et al., 2006), while the more common version European Addiction Severity Index (EuropASI) is used in Norway (Lauritsen, 2010). The two versions of ASI cover the same multidimensional areas of addiction severity, but the Swedish version contains modifications allegedly making it more closely aligned with Swedish welfare, social service, and treatment contexts, and maybe more useful in clinical practice.

A Norwegian evaluation of the EuropASI has been performed (Lauritsen, 2010). This is not a formal psychometric, but rather a narrative review. The report states that the instrument's principal contribution to clinical substance use treatment is its ability to provide a comprehensive, multidimensional assessment of patients’ needs and progress across domains that are highly relevant to recovery. Rather than focusing solely on substance use, EuropASI systematically assesses physical health, employment and economic conditions, alcohol and drug use, criminal activity, family and social relationships, and mental health, thereby supporting a holistic understanding of the patient's situation and the need for integrated interventions. The instrument can be used both at treatment entry and during follow-up, making it valuable for monitoring changes in problem severity over time and informing individualized treatment planning. According to this report, both patients and clinicians have reported positive experiences with the structured interview format and its emphasis on the patient perspective.

ASI and EuropASI are good in the sense that they not merely concentrate on drug use and addiction. They consider multiple aspects of the person's life, aspects crucial for understanding the patient's broader situation and for assessing treatment and rehabilitation needs. Drug use problems are often complex and affect many aspects of the patient's existence. However, these advantages must be balanced against important limitations. The instruments are lengthy and resource-intensive and some of the outcome measures and short assessment windows may limit its sensitivity to long-term change. Furthermore, even if no international certification is required, formal training and supervised practice are generally recommended to ensure reliable administration and scoring. Training for the EuropASI, at least in Norway, requires a 3-day introductory course for all users. Such training generates costs both in time spent and fees for the courses. However, neither ASI nor EuropASI are subject to a per-use licensing fee, and both instruments are widely available for clinical and research purposes. There may however be indirect “costs” associated with the use as ASI nor EuropASI are comprehensive and time-consuming instruments.

These costs and the training may still be worth it because such instruments may ensure that people with quite diverse backgrounds, both in level of education (unskilled, bachelor, master's degree etc) and in orientation of their profession (medicine, psychology, social worker) will have a tool that serves as a common ground of understanding, a form of benchmarking. In a Norwegian context, we have no documented number of people that have been certified in the use of EuropASI, but there are surely several hundred who have.

In clinical practice, however, EuropASI is increasingly less used in Norway. There are several reasons for this. First, as pointed out by Björk (2026), they are considered laborious and other instruments could provide similar information more efficiently. Second, they do not provide diagnosis, and other instruments must be used for this. Lastly, ASI and EuropASI do not cover other clinically important areas the health services are obliged to cover, such as mental illness, violence and suicide risk, and third-party problems (e.g., children).

Some parts of EuropASI have been used in research. It is cited that, at least in some versions, ASI and its adaptations are among the most widely used assessment instruments in addiction research and clinical evaluation internationally. It has been utilized in studies on, for example, cocaine (Crits-Christoph et al., 1999), alcohol (Ahmadi et al., 2009), and opioids (Bovasso et al., 2001). Norwegian researchers have followed with some utilization (Lauritzen & Ravndal, 2004; Ravndal & Lauritzen, 2004). However, this constitutes partial use in research, and tells us little about clinical utility.

Modern treatment places great emphasis on evidence-based practice. The fundamental principle is that clinical practice should be informed by the best available evidence rather than by tradition, authority, opinion, moral preferences, or intuition alone. This requires a critical review of scientific literature, including assessments of reliability, validity, and study design. The appeal of ASI and EuropASI derives partly from their obvious clinical relevance and face validity: they provide a structured and comprehensive assessment of multiple domains that are known to be important in understanding and treating SUDs. But intuitive appeal should not, as pointed out by Björk (2026), exempt ASI and EuropASI from scientific scrutiny. In this respect, the critique by Björk (2026) provides a valuable contribution to the discussion. When the usefulness of an instrument appears to be self-evident, researchers should evaluate their strengths and limitations.

Björk (2026) goes on to advocate the introduction of other more flexible and less extensive instruments to be used in clinical practice. This may be the way to go, especially if the instrument is both more concise and still covers clinically necessary topics. However, the sunken costs associated with the already established instrument should be taken into account, as should the fact that newer is not necessarily better. It is often easier to find fault with old solutions than to produce optimal replacements.
